# Atomic Charge Calculator II: web-based tool for the calculation of partial atomic charges

**DOI:** 10.1093/nar/gkaa367

**Published:** 2020-05-13

**Authors:** Tomáš Raček, Ondřej Schindler, Dominik Toušek, Vladimír Horský, Karel Berka, Jaroslav Koča, Radka Svobodová

**Affiliations:** CEITEC - Central European Institute of Technology, Masaryk University, Brno 625 00, Czech Republic; National Centre for Biomolecular Research, Faculty of Science, Masaryk University, Brno 602 00, Czech Republic; Faculty of Informatics, Masaryk University, Brno 602 00, Czech Republic; CEITEC - Central European Institute of Technology, Masaryk University, Brno 625 00, Czech Republic; National Centre for Biomolecular Research, Faculty of Science, Masaryk University, Brno 602 00, Czech Republic; CEITEC - Central European Institute of Technology, Masaryk University, Brno 625 00, Czech Republic; National Centre for Biomolecular Research, Faculty of Science, Masaryk University, Brno 602 00, Czech Republic; CEITEC - Central European Institute of Technology, Masaryk University, Brno 625 00, Czech Republic; National Centre for Biomolecular Research, Faculty of Science, Masaryk University, Brno 602 00, Czech Republic; Regional Centre of Advanced Technologies and Materials, Department of Physical Chemistry, Faculty of Science, Palacký University Olomouc, Olomouc 771 46, Czech Republic; CEITEC - Central European Institute of Technology, Masaryk University, Brno 625 00, Czech Republic; National Centre for Biomolecular Research, Faculty of Science, Masaryk University, Brno 602 00, Czech Republic; CEITEC - Central European Institute of Technology, Masaryk University, Brno 625 00, Czech Republic; National Centre for Biomolecular Research, Faculty of Science, Masaryk University, Brno 602 00, Czech Republic

## Abstract

Partial atomic charges serve as a simple model for the electrostatic distribution of a molecule that drives its interactions with its surroundings. Since partial atomic charges are frequently used in computational chemistry, chemoinformatics and bioinformatics, many computational approaches for calculating them have been introduced. The most applicable are fast and reasonably accurate empirical charge calculation approaches. Here, we introduce Atomic Charge Calculator II (ACC II), a web application that enables the calculation of partial atomic charges via all the main empirical approaches and for all types of molecules. ACC II implements 17 empirical charge calculation methods, including the highly cited (QEq, EEM), the recently published (EQeq, EQeq+C), and the old but still often used (PEOE). ACC II enables the fast calculation of charges even for large macromolecular structures. The web server also offers charge visualization, courtesy of the powerful LiteMol viewer. The calculation setup of ACC II is very straightforward and enables the quick calculation of high-quality partial charges. The application is available at https://acc2.ncbr.muni.cz.

## INTRODUCTION

Partial atomic charges are real numbers that model the distribution of charge density in a molecule. Thus, they provide clues to the chemical behaviour of molecules, even though they are not physically observable entities. The concept of partial atomic charges was first used in chemistry for explaining reactivity (1,2). Partial atomic charges were also adopted by computational chemistry (e.g. applications in molecular dynamics, docking, or conformational searches) (3–5) and they also became popular in chemoinformatics (e.g. descriptors for QSAR and QSPR modelling, or virtual screening) (6–8) and bioinformatics (e.g. similarity searches, or the study of mechanisms and effects connected with certain chemical actions) (9,10). Many computational approaches have been introduced for calculating them. The most reliable method for partial charge calculation is an application of quantum mechanics (QM), as recently reviewed in Cho *et al.* (11). Because QM methods are generally time-demanding, quicker non-QM empirical charge calculation approaches were developed. Specifically, the non-QM empirical methods do not consider individual electrons (or/and basis functions) in the calculations, but they work on the level of atoms. These approaches can be divided into conformationally independent, which are based on the 2D structure (so-called 2D methods; e.g. Gasteiger and Marsili's PEOE (12), MPEOE (13), KCM (14), or DENR (15)), and conformationally dependent, which are calculated from the 3D structure (so-called 3D methods; e.g. EEM (16), QEq (3), or EQeq (17)). Since non-QM empirical methods are often parameterized towards QM methods, their accuracy is comparable (see e.g. (18,19)), and due to the calculation speed, they are also applicable for biomacromolecules (10). For this reason, several non-QM empirical methods are frequently used and highly cited (e.g. PEOE > 3000 citations, EEM > 700 citations, QEq > 2000 citations), as was discovered in a literature search of the Web of Science database (https://www.webofknowledge.com/, ‘All Databases’ dataset) that we carried out on 31 March 2020. Complete results of the literature search are shown in the Supplementary Table S1.

The practical utilization of non-QM empirical charge calculation approaches brings with it three challenges:

1. Most non-QM empirical approaches are only described within an article, and their implementation is not available to the community. Only a few of the non-QM empirical methods are available as desktop software tools (OpenBabel (20), RDKit (https://www.rdkit.org/), VCharge (21), EEM-SOLVER (22), and ABEEM-SOLVER (22)), and only one non-QM empirical method (the EEM) is accessible as a web application (AtomicChargeCalculator (ACC) (23)). Despite the limited functionality of ACC, it became frequently used (∼2000 accesses per year).
2. The non-QM empirical approaches use parameters taken from physicochemical constants or QM charges. These parameters, however, are usually only optimized for specific types of molecules and not generally applicable. Therefore many parameter sets have been published, and their limitations are not easily accessible information.
3. Even though non-QM empirical approaches are much faster than QM methods, the time complexity of the conformationally dependent approaches is often O(N^3^), where N is the number of atoms in the molecule, due to solving the system of linear equations. For their application on larger molecular systems (e.g. biomacromolecules), sophisticated complexity reduction algorithms (e.g. cutoff and cover methods (23)) have to be integrated.

In this article, we address all these challenges, and we provide Atomic Charge Calculator II, an update to ACC, which includes these key innovations:

- Implementation of 17 charge calculation approaches, including the highly cited (QEq, EEM), the recently published (EQeq, EQeq+C), and the old but still often used (PEOE). Where applicable, the approaches that involve solving linear equation systems use cutoff and cover methods (23) for the fast processing of large macromolecular structures. If the approaches utilize parameters, all the published parameter sets were collected from the literature and integrated into ACC II. The list of all parameters included in ACC II is available in the Supplementary Table S2.
- The visualization of charges uses the powerful LiteMol viewer (24), which offers several viewing options as well as the manipulation of computation results.
- The calculation setup is very straightforward and enables the quick calculation of high-quality charges.

## DESCRIPTION OF THE WEB SERVER

ACC II is an interactive web application for the calculation of partial atomic charges via non-QM empirical charge calculation approaches and for the visualization of these charges. ACC II is composed of a frontend and a backend. The frontend is a modern web application written in JavaScript using the Bootstrap library. Its first function is to read the user input that consists of molecular structure(s) and computation settings (e.g. one of the charge calculation methods that are integrated into the backend). Its second purpose is to present the output, i.e. calculated charges. These charges are available as downloadable data files, and can also be visualized via the LiteMol viewer, which is part of the ACC II frontend. The backend is a Python Flask application. All the computations of charges are carried out by the core C++ application, which integrates 17 non-QM empirical charge calculation approaches. It also includes the implementation of cutoff and cover methods (23) for the fast solving of linear equation systems. All three parts of ACC II are available on GitHub under the MIT license: the frontend and backend are available at https://github.com/krab1k/AtomicChargeCalculator2, while the core is available at https://github.com/krab1k/ChargeFW2.

### Non-QM empirical charge calculation approaches in ACC II

**Figure 1.**
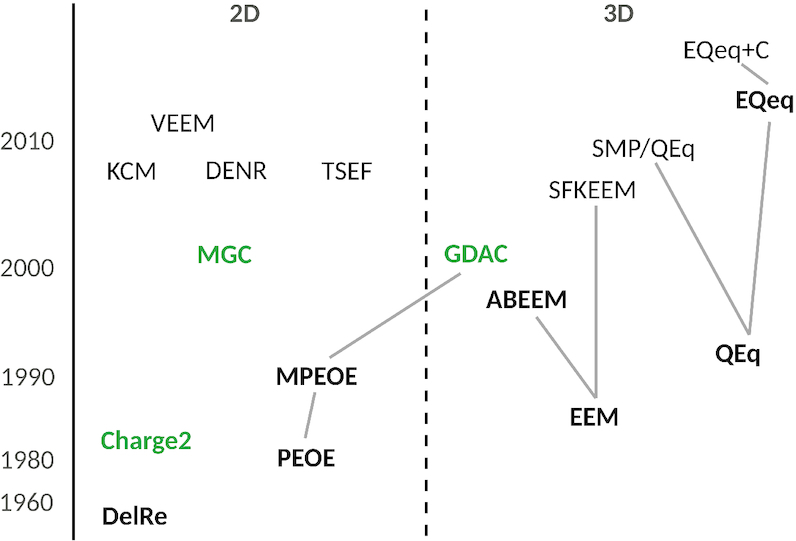
Overview of non-QM empirical charge calculation methods integrated into ACC II. The left axis shows the year of a method's publication. The methods are divided into 2D and 3D approaches. When two methods are connected by a line, the upper one was developed based on the lower one (e.g. ABEEM and SFKEEM are successors of EEM). The methods written in bold were cited more than 100 times, while the methods in green were cited more than 20 times. The citation numbers were obtained via a literature search of the Web of Science database (https://www.webofknowledge.com/, ‘All Databases’ dataset) that we carried out on 31 March 2020. Citation count of each method implemented in ACC II is provided in the Supplementary Table S1.

ACC II integrates nine conformationally independent (2D) methods (PEOE (12), DelRe (25), MPEOE (13), Charge2 (26), MGC (27,28), KCM (14), DENR (15), TSEF (15), and VEEM (29)) and eight conformationally dependent (3D) methods (EEM (16), ABEEM (30), SFKEEM (31), QEq (3), SMP/QEq (32), EQeq (17), EQeq+C (33), and GDAC (34)). An overview of these methods is depicted in Figure 1, which also shows relationships between the methods (i.e. if two methods are connected by a line, the upper is a successor of the lower), their division into 2D and 3D approaches, and the year of their publication.

The principles and theoretical basis of all these methods (including their quality criteria) are described in the Short description of the methods in the Supplementary data.

### ACC II workflow

The procedure for using the ACC II application involves six steps: (i) uploading the structure(s), (ii) internal validation, (iii) selecting the non-QM empirical method and its parameter set, (iv) executing the selected method, (v) visualizing the computed charges, (vi) downloading the computed charges.

*(i) Uploading the structure(s)*. The first step is to upload the molecular structure for which the charges will be calculated. The structure can be provided in SDF, MOL2, PDB, or mmCIF file formats. ACC II is also able to accept more than one structure. In this case, the accepted formats are the same, but the input files have to be compressed into one input archive, which can be in zip or tar.gz format.

*(ii) Internal validation*. The input files are then validated. Specifically, ACC II tests whether the files are in one of the supported formats and whether they contain the necessary information for the description of a molecular structure (i.e. coordinates of the atoms, definition of bonds in the case of small molecules). If the input files do not pass the validation procedure, the user is informed about detected problems. The most common one is that the input file does not conform to the standard of a particular file format.

*(iii) Selecting the non-QM empirical method and its parameter set*. After reading the input molecule(s), ACC II first detects non-QM empirical methods which can be executed on the user's data. Specifically, each non-QM empirical approach has one or more parameter sets. Some non-QM empirical approaches use just the tabular values of physicochemical constants as parameters. But most of the methods require more complex parameter sets, also containing parameters for individual elements (e.g. if the parameter set is focused on proteins, it contains parameters for C, H, O, N, and S, but it can lack parameters for Cl, Br, I, Si, etc.). Such a method can only be executed on a molecule composed of elements that are parameterized in at least one parameter set belonging to the method. Note that the methods that do not need parameters for individual elements cover every molecule. If a set of molecules is provided by a user, they can only use those approaches which cover all of the input molecules. Please note that it makes no sense to use multiple parameter sets on a single set of input molecules, since the computed charges will not be comparable. Methods that can be used for the specific input data set are further denoted as ‘applicable methods’.

The users have two ways of selecting a non-QM empirical charge calculation approach from the applicable methods: they can use the automatic setup via the ‘Compute charges’ button or select a method themselves by pressing the ‘Setup computation’ button.

If the users select the calculation method themselves, they can not only choose the method, but also its parameter set where applicable (e.g. if more than one parameter set was published). On the ‘Computation settings’ web page, each approach and each parameter set are supplemented by a reference to the publication in which it was described.

If the user prefers the automatic setup, ACC II selects the approach that was documented as being the most suited from the available methods. Details about the selection process of the most suited charge calculation method are described in the ACC II online documentation (https://acc2.ncbr.muni.cz/static/manual.pdf). If the approach has more parameter sets, the one that is the most suitable for the specific input molecules is selected (e.g. a parameter set specialized on drug-like molecules is used for small organic molecules). The list of parameter sets included in ACC II is available in the Supplementary Table S2.

*(iv) Executing the selected method*. The selected charge calculation approach is executed on the backend for each input molecular structure. Each computation on the backend has two inputs: A user-provided molecular structure and the selected parameter set. If the approach integrates cutoff and cover methods for solving a linear equation system and the molecular structure has >20 000 atoms but <80 000 atoms, the cutoff method is utilised. If the number of atoms is equal to or higher than 80 000, the cover method is utilised.

**Figure 2.**
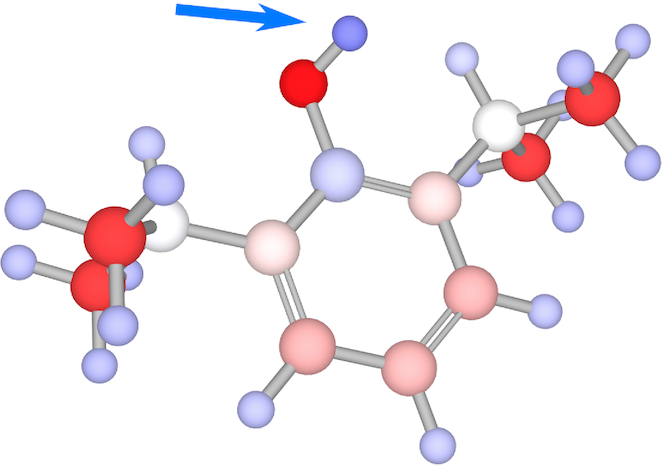
Partial atomic charges in propofol. The phenol hydrogen is marked with a blue arrow. The partial atomic charges were calculated by EEM.

**Figure 3.**
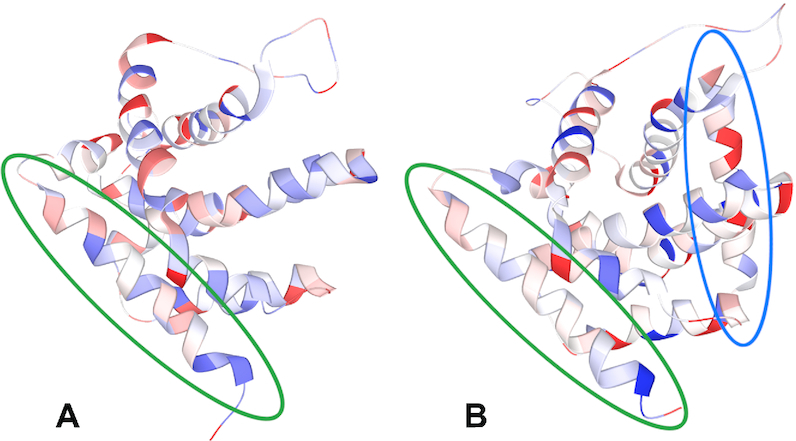
(**A**) Inactive BAX (PDB ID 1f16). (**B**) Activated BAX (PDB ID 2k7w). An activator is marked with a **blue oval**, the C domain is marked with a **green oval**. The C domain of activated BAX is depolarized – it is mainly white or whitish in colour. This depolarization causes the C domain to be released and penetrate the mitochondrial membrane and initiate apoptosis. The partial atomic charges were calculated by EEM.

**Figure 4.**
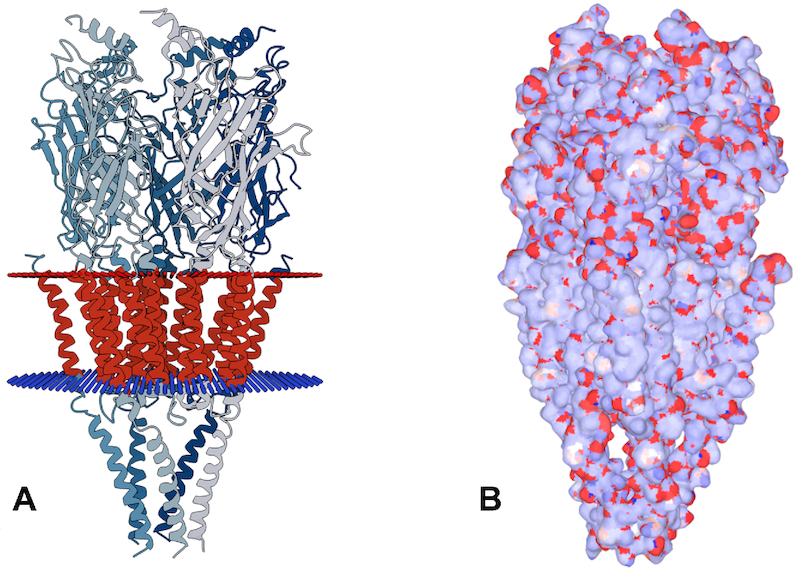
Structure of nicotinic acetylcholine receptor (PDB ID 2bg9): (**A**) Scheme showing the receptor (in grey) passing the membrane (in red). The figure was taken from RCSB PDB. (**B**) Partial atomic charges (from ACC II) visualized on the surface of the receptor structure showing distinct areas: Nonpolar transmembrane part (mostly white due to charge being around zero) and polar surface of extracellular and cytoplasmic parts (with a mosaic of blue positive and red negative charges). The partial atomic charges were calculated by EEM.

*(v) Visualizing the computed charges*. The calculated charges can be presented to the user via the LiteMol viewer that is integrated into ACC II (specifically, in its ‘Computation results’ web page). Three visualization models can be used: balls and sticks model, cartoon model, and surface model. All three visualization models can be coloured using the values of the computed charges. In the balls and sticks model (see Figure 2), the balls are coloured directly according to the partial atomic charge values. In the cartoon model (see Figure 3), the helices, sheets and tubes between them are divided into regions that represent individual amino acids. Each region is coloured according to the sum of atomic charges that belongs to a particular amino acid. In the surface model (see Figure 4B), the surface is divided into parts that belong to individual surface atoms. Each part of the surface is then coloured according to the partial atomic charge of the atom it represents.

The colour scale spans from blue through white to red. Negative charges are red (the more negative the value of the charge, the more intense the colour) and positive charges are blue (the more positive the value of the charge, the more intense the colour). The closer the value of the charge is to zero, the closer its colour is to white. A user can select the relative colour scale that spans from the lowest to the highest charge value in the visualized structure, or an absolute colour scale that spans from a user-defined value to another user-defined value).

*(vi) Downloading the computed charges*. Partial atomic charges calculated using ACC II can be downloaded in PQR, MOL2, and plaintext file formats. ACC II provides one ZIP file containing charges for all input molecules in relevant output formats (PQR for proteins, MOL2 for small molecules, plaintext for both).

### Limitations

ACC II currently has a few limitations: It includes only non-QM empirical charge calculation methods (not QM), which are fully automated (no hand-tuning required) and which methodology is sufficiently described in its publication. The size of the input file is limited to 10 MB. The cartoon visualization model is only available when the input file is in the PDB or mmCIF format (i.e. formats containing information about amino acids and other residues). Non-QM empirical approaches that require parameters for individual atoms can only process molecules for which at least one of their parameter sets covers these molecules. The cutoff and cover methods for the fast solving of linear equation systems are integrated into EEM, SFKEEM, QEq, SMP/QEq, EQeq, EQeq+C. Other methods that involve linear equation systems employ different schemes for which the cutoff and cover methods are not directly applicable. Due to a limitation of LiteMol, the computation result cannot be visualized when an input molecule was provided in an mmCIF file that lacks the ‘_atom_sites’ record.

## RESULTS AND DISCUSSION

We provide three examples which demonstrate possible uses of the ACC II web application. The interactive form of these examples is presented on the ACC II webpage. Files with structures from these examples are available in the Supplementary data.

### Example I – dissociating hydrogens from phenols

In the first example, we show a charge calculation for seven phenolic drug compounds (see Table 1), described in DrugBank. We obtained their structures from the PubChem database and calculated their partial atomic charges using ACC II (automatic setup). The results of the calculation are available on the ACC II web page. In this example, we would also like to provide a preview of charge utilization—an application in the field of acid dissociation study. Acid dissociation is a reaction in which a molecule releases a hydrogen atom. The ability of the molecule to release the hydrogen is described by its acid dissociation constant (*K*_a_) and its negative logarithm (p*K*_a_). The relation between the charge of the dissociating hydrogen and p*K*_a_ is well known and often used for p*K*_a_ prediction (6,7,35). In our example, we focused on this relation. The dissociating hydrogen is a part of the phenolic OH group. For each of our seven compounds, we obtained the p*K*_a_ value (from (7)) and the charge on the phenolic hydrogen and summarized these values in Table 1. It can be seen from Table 1 that there is a clear dependence between p*K*_a_ and the charge on the phenolic hydrogen. Specifically, the higher the p*K*_a_ (dissociation requires higher pH), the lower charge the hydrogen has. More details about the relationship between p*K*_a_ and charges in phenols and its application can be found here (7).

**Table 1.** Summary information about phenolic drug compounds

Name of the compound	DrugBank ID	PubChem CID	p*K*_a_	Charge on phenolic H
2,4-Dinitrophenol	DB04528	1493	4.09	0.467
4-Nitrophenol	DB04417	980	7.15	0.430
2-Chlorophenol	DB03110	7245	8.56	0.405
3-Chlorophenol	DB01957	7933	9.12	0.393
*m*-Cresol	DB11143*	342	10.10	0.379
*o*-Cresol	DB11143*	335	10.30	0.376
Propofol	DB00818	4943	11.10	0.350

*DrugBank ID links to the Cresol mixture.

Note: The partial atomic charges were calculated by EEM.

### Example II – apoptotic protein activation

In the second example, we would like to show an application of charges in protein research. Specifically, we focus on the apoptotic protein BAX in its inactive and activated forms. We obtained the structures from Protein Data Bank (the inactive form has PDB ID 1f16 and the active PDB ID 2k7w) and calculated their partial atomic charges using ACC II (automatic setup). In this comparison, the BAX protein initiates apoptosis in the following way: its C domain (marked green in Figure 3) releases and penetrates the mitochondrial membrane (10). The release is enabled by an activator (Figure 3B, blue oval), which causes a redistribution of partial atomic charges, a depolarization of the C domain and discharge of electrostatic forces binding the domain (see Figure 3). Partial atomic charges provide us with a clue to understanding the BAX activation mechanism. This mechanism is described in detail in the article (10).

### Example III – transmembrane protein

In the third example, we show a charge calculation for a large transmembrane protein – the nicotinic acetylcholine receptor. This receptor passes the cell membrane (see Figure 4A) and serves as an ion channel (36). We obtained its structure from Protein Data Bank (PDB ID: 2bg9), added missing hydrogens via WHAT IF (37)) and calculated the partial atomic charges using ACC II (automatic setup). The visualization of partial charges on the surface (see Figure 4B) highlights the difference between the nonpolar transmembrane part (mostly white due to charge around zero) and the polar surface of the extracellular and cytoplasmic parts (with a mosaic of blue positive and red negative charges). The comparison demonstrates that this charge distribution agrees with the receptor membrane position reported in the literature (36).

## CONCLUSION

In this article, we presented ACC II, a novel web application for the calculation of partial atomic charges using all the main non-QM empirical approaches and for all types of molecules including biomacromolecules. ACC II also allows the visualization of charges via three main charge visualization models. The web application is easy to use and is platform-independent. Viewing results and manipulations of them are fully interactive. All results of ACC II can be downloaded in various formats (PQR, MOL2, and plaintext format). Documentation explaining the methodology and examples is provided on the webpage of ACC II.

ACC II is freely available at https://acc2.ncbr.muni.cz with no login requirement.

## Supplementary Material

gkaa367_Supplemental_Files

## References

[1] Berzelius J. Erste fortsetzung des versuchs, die bestimmten und einfachen Verhältnisse aufzufinden, nach welchen die Bestandtheile der unorganischen Natur mit einander verbunden sind. Ann. Phys. 1811; 38:161–226.

[2] Mulliken R.S. Electronic population analysis on LCAO-MO molecular wave functions. I. J. Chem. Phys. 1955; 23:1833–1840.

[3] Rappé A.K., Goddard W.A. Charge equilibration for molecular dynamics simulations. J. Phys. Chem. 1991; 95:3358–3363.

[4] Park H., Lee J., Lee S. Critical assessment of the automated AutoDock as a new docking tool for virtual screening. Proteins Struct. Funct. Bioinforma. 2006; 65:549–554. 10.1002/prot.21183 16988956

[5] Vainio M.J., Johnson M.S. Generating conformer ensembles using a multiobjective genetic algorithm. J. Chem. Inf. Model. 2007; 47:2462–2474. 17892278 10.1021/ci6005646

[6] Gross K.C., Seybold P.G., Hadad C.M. Comparison of different atomic charge schemes for predicting pKa variations in substituted anilines and phenols. Int. J. Quantum Chem. 2002; 90:445–458.

[7] Svobodová Vařeková R., Geidl S., Ionescu C.-M., Skřehota O., Kudera M., Sehnal D., Bouchal T., Abagyan R., Huber H.J., Koča J. Predicting pKa values of substituted phenols from atomic charges: comparison of different quantum mechanical methods and charge distribution schemes. J. Chem. Inf. Model. 2011; 51:1795–1806. 21761919 10.1021/ci200133w

[8] Bissantz C., Folkers G., Rognan D. Protein-based virtual screening of chemical databases. 1. Evaluation of different docking/scoring combinations. J. Med. Chem. 2000; 43:4759–4767. 11123984 10.1021/jm001044l

[9] Holliday J.S., Jelfs S.P., Willett P., Gedeck P. Calculation of intersubstituent similarity using R-group descriptors. J. Chem. Inf. Comput. Sci. 2003; 43:406–411. 12653502 10.1021/ci025589v

[10] Ionescu C.-M., Svobodová Vařeková R., Prehn J.H.M., Huber H.J., Koča J. Charge profile analysis reveals that activation of pro-apoptotic regulators bax and bak relies on charge transfer mediated allosteric regulation. PLoS Comput. Biol. 2012; 8:e1002565. 22719244 10.1371/journal.pcbi.1002565 PMC3375244

[11] Cho M., Sylvetsky N., Eshafi S., Santra G., Efremenko I., Martin J.M.L. The atomic partial charges arboretum: trying to see the forest for the trees. ChemPhysChem. 2020; 21:688–696. 32052532 10.1002/cphc.202000040 PMC7317385

[12] Gasteiger J., Marsili M. A new model for calculating atomic charges in molecules. Tetrahedron Lett. 1978; 34:3181–3184.

[13] No K.T., Grant J.A., Scheraga H.A. Determination of net atomic charges using a modified partial equalization of orbital electronegativity method. 1. Application to neutral molecules as models for polypeptides. J. Phys. Chem. 1990; 94:4732–4739.

[14] Yakovenko O., Oliferenko A.A., Bdzhola V.G., Palyulin V.A., Zefirov N.S. Kirchhoff atomic charges fitted to multipole moments: implementation for a virtual screening system. J. Comput. Chem. 2008; 29:1332–1343. 18172839 10.1002/jcc.20892

[15] Shulga D.A., Oliferenko A.A., Pisarev S.A., Palyulin V.A., Zefirov N.S. Fast tools for calculation of atomic charges well suited for drug design. SAR QSAR Environ. Res. 2008; 19:153–165. 18311641 10.1080/10629360701844142

[16] Mortier W.J., Ghosh S.K., Shankar S. Electronegativity equalization method for the calculation of atomic charges in molecules. J. Am. Chem. Soc. 1986; 108:4315–4320.

[17] Wilmer C.E., Kim K.C., Snurr R.Q. An extended charge equilibration method. J. Phys. Chem. Lett. 2012; 3:2506–2511. 26292141 10.1021/jz3008485

[18] Geidl S., Bouchal T., Raček T., Svobodová Vařeková R., Hejret V., Křenek A., Abagyan R., Koča J. High-quality and universal empirical atomic charges for chemoinformatics applications. J. Cheminform. 2015; 7:59. 26633997 10.1186/s13321-015-0107-1 PMC4667495

[19] Raček T., Pazúriková J., Svobodová Vařeková R., Geidl S., Křenek A., Falginella F.L., Horský V., Hejret V., Koča J. NEEMP: software for validation, accurate calculation and fast parameterization of EEM charges. J. Cheminform. 2016; 8:57. 27803746 10.1186/s13321-016-0171-1 PMC5067907

[20] O’Boyle N.M., Banck M., James C.A., Morley C., Vandermeersch T., Hutchison G.R. Open Babel: an open chemical toolbox. J. Cheminform. 2011; 3:33. 21982300 10.1186/1758-2946-3-33 PMC3198950

[21] Gilson M.K., Gilson H.S.R., Potter M.J. Fast assignment of accurate partial atomic charges: an electronegativity equalization method that accounts for alternate resonance forms. J. Chem. Inf. Comput. Sci. 2003; 43:1982–1997. 14632449 10.1021/ci034148o

[22] Svobodová Vařeková R., Koča J. Optimized and parallelized implementation of the electronegativity equalization method and the atom-bond electronegativity equalization method. J. Comput. Chem. 2006; 27:396–405. 16381078 10.1002/jcc.20344

[23] Ionescu C.-M., Sehnal D., Falginella F.L., Pant P., Pravda L., Bouchal T., Svobodová Vařeková R., Geidl S., Koča J. AtomicChargeCalculator: interactive web-based calculation of atomic charges in large biomolecular complexes and drug-like molecules. J. Cheminform. 2015; 7:50. 26500704 10.1186/s13321-015-0099-x PMC4613891

[24] Sehnal D., Deshpande M., Svobodová Vařeková R., Mir S., Berka K., Midlik A., Pravda L., Velankar S., Koča J. LiteMol suite: Interactive web-based visualization of large-scale macromolecular structure data. Nat. Methods. 2017; 14:1121–1122. 29190272 10.1038/nmeth.4499

[25] Del Re G. A simple MO-LCAO method for the calculation of charge distributions in saturated organic molecules. J. Chem. Soc. 1958; 11:4031–4040.

[26] Abraham R.J., Hudson B. Approaches to charge calculations in molecular mechanics. J. Comput. Chem. 1982; 3:407–416.

[27] Oliferenko A.A., Palyulin V.A., Neiman A.V., Zefirov N.S. A new topological model for the calculation of partial atomic charges. Dokl. Chem. 2000; 375:281–284.

[28] Oliferenko A.A., Palyulin V.A., Pisarev S.A., Neiman A.V., Zefirov N.S. Novel point charge models: reliable instruments for molecular electrostatics. J. Phys. Org. Chem. 2001; 14:355–369.

[29] Wu Y.-X., Cao C.-Z., Yuan H. Equalized electronegativity based on the valence electrons and its application. Chinese J. Chem. Phys. 2011; 24:31–39.

[30] Yang Z.-Z., Wang C.-S. Atom-bond electronegativity equalization method. 1. Calculation of the charge distribution in large molecules. J. Phys. Chem. A. 1997; 101:6315–6321.

[31] Chaves J., Barroso J.M., Bultinck P., Carbó-Dorca R. Toward an alternative hardness kernel matrix structure in the Electronegativity Equalization Method (EEM). J. Chem. Inf. Model. 2006; 46:1657–1665. 16859297 10.1021/ci050505e

[32] Zhang M., Fournier R. Self-consistent charge equilibration method and its application to Au_13_Na_n_ (n = 1, 10) clusters. J. Phys. Chem. A. 2009; 113:3162–3170. 19320517 10.1021/jp8063273

[33] Martin-Noble G.C., Reilley D., Rivas L.M., Smith M.D., Schrier J. EQeq+C: an empirical bond-order-corrected extended charge equilibration method. J. Chem. Theory Comput. 2015; 11:3364–3374. 26575770 10.1021/acs.jctc.5b00037

[34] Cho K.H., Kang Y.K., No K.T., Scheraga H.A. A fast method for calculating geometry-dependent net atomic charges for polypeptides. J. Phys. Chem. B. 2001; 105:3624–3634.

[35] Liptak M.D., Gross K.C., Seybold P.G., Feldgus S., Shields G.C. Absolute pKa determinations for substituted phenols. J. Am. Chem. Soc. 2002; 124:6421–6427. 12033873 10.1021/ja012474j

[36] Unwin N. Refined structure of the nicotinic acetylcholine receptor at 4 Å resolution. J. Mol. Biol. 2005; 346:967–989. 15701510 10.1016/j.jmb.2004.12.031

[37] Vriend G. WHAT IF: a molecular modeling and drug design program. J. Mol. Graph. 1990; 8:52–56. 2268628 10.1016/0263-7855(90)80070-v

