# Supplementary material for: Atomic Charge Calculator II: web-based tool for the calculation of partial atomic charges
Source: Nucleic Acids Res. 2020 May 13;48(W1):W591–6. doi: 10.1093/nar/gkaa367 (PMC7319571; doi:10.1093/nar/gkaa367)
Supplement: gkaa367_Supplemental_Files [file gkaa367_supplemental_files.zip › Short description of the methods.pdf]

Short description of the methods

This section shortly describes the main idea of each method implemented in Atomic Charge Calculator II. Methods here are ordered according to the publication date.

For clarity, we use unifying naming scheme (which may differ from the one used in the original publication); the symbols used throughout the chapter: q_i stands for charge on ith atom, Q is the total molecular charge, N is the number of atoms in a molecule and $R_{i,j}$ represents the Euclidean distance between atoms i and j.

DelRe

Methods of Del Re [1] starts with the definition of a linear system in the form:

$$\delta_i = \delta_i^0 + \sum_j \gamma_{i,j}\delta_j \tag{1}$$

where j iterates over atoms bonded to i. δ_i^0 is an atom parameter, whereas $\gamma_{i,j}$ is a bond parameter. Solving for δ_i allow us to derive bond charges:

$$q_{i,j} = \frac{\delta_i - \delta_j}{2\epsilon_{i,j}} \tag{2}$$

where $\epsilon_{i,j}$ is another bond parameter. Finally, charge for each atom is computed as the sum of all involved bond charges.

PEOE

Partial equalization of orbital electronegativity [2, 3] is an iterative scheme in which the charges are moved along the bonds from the more electropositive atom to the more electronegative one. The amount of charge shifted is proportional to the difference of the electronegativities of the bonding partners. As effective electronegativity is defined here as a function of charge:

$$\chi_i^\alpha = A_i + B_i q^\alpha + C_i (q_i^\alpha)^2 \tag{3}$$

its value for each atom must be recomputed as it enters the next iteration.

The main idea of a charge transfer is expressed through the following equation:

$$q_i^\alpha = \left(\sum_j \frac{\chi_j^\alpha - \chi_i^\alpha}{D_i} + \sum_k \frac{\chi_i^\alpha - \chi_k^\alpha}{D_k} \right) \cdot \left(\frac{1}{2}\right)^\alpha \tag{4}$$

where j are atoms bonded to atom i with higher electronegativity and k are atoms bonded to atom i with lower electronegativity.

Since the generated charges produce an electrostatic field which further hinders the charge transfer, the dampening factor $(1/2)^\alpha$ was introduced to account for that fact. Usually, six iterations of 3 followed by 4 are necessary for charges to converge.

Finally, total atomic charge q_i is the sum of charge transfers across all the iterations:

$$q_i = \sum_\alpha q_i^\alpha \tag{5}$$

Charge2

Charge2 [4] is an iterative method in which charge increments from neighbor atoms are added to a central one.

$$q_i = q_i(\alpha) + q_i(\beta) + q_i(\gamma) \tag{6}$$

$$q_i(\alpha) = \sum_j \frac{\chi_j - \chi_i}{a} \tag{7}$$

$$q_i(\beta) = \sum_k \frac{(\chi_k - \chi_H)P_i}{b} \tag{8}$$

$$q_i(\gamma) = \sum_l \frac{(\chi_l - \chi_H)P_i}{bc} \tag{9}$$

$$\tag{10}$$

where

$$P_i = P_i^0 \left(1 + \alpha(q_i^0 - q_i)\right) \tag{11}$$

and j, k and l represents atoms one, two or three bonds apart from atom i, a, b, c and P^0 are atom parameters, q^0 is a formal charge, χ_H is an electronegativity of hydrogen, and α is a common parameter.

EEM

Contrary to the partial electronegativity equalization methods like PEOE or MPEOE, full electronegativity equalization is fundamental to the Mortier's Electronegativity Equalization Method [5].

According to the Sanderson's principle, the electronegativity of each atom gets equalized when atoms bond to form a molecule:

$$\overline{\chi} = \chi_1 = \ldots = \chi_N \tag{12}$$

The electronegativity of an atom in a molecule is expressed as:

$$\chi_i = A_i + B_i q_i + \sum_{i \neq j} \frac{q_j}{R_{i,j}} \tag{13}$$

where

$$A_i = \chi_i^0 + \Delta\chi_i \tag{14}$$

$$B_i = 2(\eta_i^0 + \Delta\eta_i) \tag{15}$$

χ^0 is an electronegativity of an isolated atom, η^0 is a hardness of an isolated atom. Δ symbols represent corrections for the molecular environment.

Finally, charge conservation principle holds:

$$Q = \sum_i q_i \tag{16}$$

Rewriting 13 for every atom in molecule subject to 12 and 16 yields a system of $N + 1$ linear equations:

$$\begin{bmatrix} B_1 & R_{1,2}^{-1} & \cdots & R_{1,N}^{-1} & 1 \\ R_{2,1}^{-1} & B_2 & \cdots & R_{2,N}^{-1} & 1 \\ \vdots & \vdots & \ddots & \vdots & \vdots \\ R_{N,1}^{-1} & R_{N,2}^{-1} & \cdots & B_N & 1 \\ 1 & 1 & \cdots & 1 & 0 \end{bmatrix} \cdot \begin{bmatrix} q_1 \\ q_2 \\ \vdots \\ q_N \\ -\overline{\chi} \end{bmatrix} = \begin{bmatrix} -A_1 \\ -A_2 \\ \vdots \\ -A_N \\ Q \end{bmatrix} \tag{17}$$

MPEOE

Modified Partial Equalization of Orbital Electronegativity [6] differs from original PEOE by expressing electronegativity as a linear function of charge, so that 3 is modified to:

$$\chi_i^\alpha = A_i + B_i q^\alpha \tag{18}$$

Other than that, the dampening factor $(1/2)^\alpha$ is a now considered as a bond type dependent parameter $f_{x,y}$ changing 4 to:

$$q_i^\alpha = \sum_j \frac{\chi_j^\alpha - \chi_i^\alpha}{D_i} f_{i,j}^\alpha + \sum_k \frac{\chi_i^\alpha - \chi_k^\alpha}{D_k} f_{i,k}^\alpha \tag{19}$$

QEq

Charge Equilibration (QEq) [7] is similar to EEM. However, originally, it was meant as an iterative scheme as parameter $J_{i,i}$ for hydrogen was defined to be charge-dependent.

$$\chi_i = \chi_i^0 + J_{i,i}^0 q_i + \sum_{i \neq j} J_{i,j} q_j \tag{20}$$

In the original publication values for the Coulomb repulsion term $J_{i,j}$ were obtained using *ab-initio* calculations. To simplify the process, several empirical terms were developed to substitute $J_{i,j}$ with some simple expression.

The system of linear equations is constructed and solved for q similarly to the one in EEM.

ABEEM

Atom-bond Electronegativity Equalization Method [8] extends original EEM to also include bond electronegativities into the equalization scheme. Electronegativity of atom i is expressed as:

$$\chi_i = A_i + B_i q_i + C_i \sum_{i-j} q_{i-j} + k \sum_{i \neq j} \frac{q_j}{R_{i,j}} + k \sum_{k-l \neq i-j} \frac{q_{k-l}}{R_{i,k-l}} \tag{21}$$

where A, B and C are atom parameters, k is a common parameter, q_{i-j} denotes the charge on the bond $i-j$. Distance to a bond is computed to its center proportional to the covalent radius of the constituent atoms. Electronegativity of a bond $i-j$ has the following form:

$$\chi_{i-j} = A_{i-j} + B_{i-j} q_{i-j} + C_{i-j,i} q_i + D_{i-j,j} q_j + k \sum_{k \neq i,j} \frac{q_k}{R_{i-j,k}} + k \sum_{k-l \neq i-j} \frac{q_{k-l}}{R_{i-j,k-l}} \tag{22}$$

where A, B, C and D are bond parameters and k is a common parameter.

Solving the system of linear equations provides us with the atomic and bond charges. The bond charges are then added onto the constituent atoms proportionally to their covalent radii yielding the final atomic charges.

GDAC

Further modification of MPEOE method is coined Geometry-dependent Atomic Charges [9]. GDAC modifies the dampening term $f_{x,y}$ to be geometry dependent:

$$f_{x,y} = 1 - \frac{R_{x,y}}{R_x^{vdw} + R_y^{vdw}} \tag{23}$$

where R_x^{vdw} stands for the van der Waals radius of atom x.

MGC

Molecular Graph Charge model (MGC) [10, 11] uses molecular graph representation of the molecule as it is inspired by electrical circuits and Kirchhoff's current laws. Therefore, no atomic coordinates are employed.

MGC constructs auxiliary matrix $\boldsymbol{S}$ in the following way:

$$\boldsymbol{S} = -\boldsymbol{A} + \boldsymbol{D} + \boldsymbol{I} \tag{24}$$

where $\boldsymbol{A}$ is a connectivity matrix, $\boldsymbol{D}$ represents diagonal degree matrix and $\boldsymbol{I}$ is a standard identity matrix.

Equalized electronegativities are obtained from those of isolated atoms as a solution to the system of linear equations.

$$\boldsymbol{S}\boldsymbol{\chi} = \boldsymbol{\chi}^0 \tag{25}$$

Finally, partial atomic charges are computed as a difference between equalized and standard electronegativities of respective atoms, divided by the average electronegativity χ_M (geometric average).

$$\boldsymbol{q} = \frac{\boldsymbol{\chi} - \boldsymbol{\chi}^0}{\boldsymbol{\chi}_M} \tag{26}$$

SFKEEM

Selfconsistent Functional Kernel Equalized Electronegativity Method [12] develops on EEM's main idea. However, it incorporates different hardness matrix. The electronegativity equalization principle in SFKEEM is expressed as:

$$\chi_i = A + 2B_i q_i + \sum_{i \neq j} 2\sqrt{B_i B_j} \operatorname{sech}(\sigma R_{i,j}) \tag{27}$$

where A, B and σ are empirical parameters and sech is a hyperbolic secant function.

KCM

Kirchhoff Charge Model [13] builds a Laplacian matrix $\boldsymbol{L}$ as:

$$\boldsymbol{L} = \boldsymbol{B}^T \boldsymbol{W} \boldsymbol{B} \tag{28}$$

where $\boldsymbol{B}$ is an incidence matrix and $\boldsymbol{W}$ is a diagonal "softness" matrix with elements $w_{i,i} = 1/(\eta_i + \eta_j)$, where η stands for hardness of an atom.

Atomic charges are derived using the following expression:

$$\boldsymbol{q} = (\boldsymbol{L}^{-1} - \boldsymbol{I})\boldsymbol{\chi}^{\mathbf{o}} \tag{29}$$

where $\boldsymbol{L}^{-1}$ is an inverse of $\boldsymbol{L}$ and $\boldsymbol{\chi}^{\mathbf{o}}$ represents a vector of electronegativities of isolated atoms.

DENR

Dynamic electronegativity relaxation [14] is an iterative 2D scheme in which charges are derived using a Laplacian matrix:

$$\boldsymbol{q}^{(n+1)} = (\boldsymbol{I} + c\Delta t \cdot \boldsymbol{B}_0)^{-1} \cdot (\boldsymbol{q}^{(n)} - c\Delta t \cdot \boldsymbol{a}_0) \tag{30}$$

where $\boldsymbol{B}_0 = \boldsymbol{L}\boldsymbol{\eta}_0$ and $\boldsymbol{a}_0 = \boldsymbol{L}\boldsymbol{\chi}_0$. Note that $\boldsymbol{\eta}_0$ is a diagonal matrix of atomic hardnesses.

TSEF

Topologically Symmetrical Energy Function [14] has electronegativity equalization principle as its base but changes off-diagonal term to include bond distance rather than Euclidean distance making TSEF conformationally independent.

$$\phi_{i,j} = \alpha \cdot K(\mathrm{MDP}_{i,j}) \cdot \frac{1}{0.84 \cdot \mathrm{MDP}_{i,j} + 0.46} \tag{31}$$

where MDP stands for *minimal distance path*, i.e., minimal number of bonds between two atoms, K is parameter and α is a unit conversion factor.

SMP/QEq

Self-Consistent Charge Equilibration Method [15] builds upon the idea of the original QEq, electronegativity of an atom is formalized as a function of charge, thus the whole scheme is a iterative one. The main equation follows:

$$\chi_i(q_i) = A_i + 2\lambda(q_i)q_i + \sum_{i \neq j} J_{i,j} q_j \tag{32}$$

where

$$\lambda(q_i) = B_i + C_i q_i + D_i (q_i)^2 \tag{33}$$

and

$$J_{i,j} = \left(\frac{1}{(2\sqrt{B_i B_j})^3} + R_{i,j}^3 \right)^{-1/3} \tag{34}$$

where A, B, C and D are atom parameters.

VEEM

Valence electrons equilibration method [16] calculates atomic charges based on the number of valence electrons of individual atoms and atomic groups.

First, equalized electronegativity is calculated for the whole molecule:

$$\chi_{ve} = \frac{\sum_i \chi_i N_{ve,i}}{\sum_i N_{ve,i}} \tag{35}$$

where χ_i is an electronegativity of isolated atom i; $N_{ve,i}$ stands for the number of valence electrons of atom i.

Finally, the partial atomic charge of atom i is computed as:

$$q_i = N_{ve,i} \frac{\chi_{ve} - \chi_i}{\chi_{ve}} \tag{36}$$

EQeq

Extended charge equilibration method [17] builds upon original QEq scheme, which is modified to take the following form (the simplest, non-periodic case without different charge centers):

$$\chi_i = \chi_i^0 + J_i^0 q_i + \frac{K}{2} \sum_{i \neq j} q_j \left(\frac{1}{R_{i,j}} + O_{i,j} \right) \tag{37}$$

where

$$\chi_i^0 = \frac{IP_i + EA_i}{2} \tag{38}$$

$$J_i^0 = IP_i - EA_i \tag{39}$$

K is a constant; IP and EA stand for ionization potential and electron affinity, respectively, and

$$O_{i,j} = \exp\left(-\frac{J_{i,j}^2 R_{i,j}^2}{K^2} \right) \cdot \left(\frac{J_{i,j}}{K} - \frac{J_{i,j}^2 R_{i,j}}{K^2} - \frac{1}{R_{i,j}} \right) \tag{40}$$

where $J_{i,j}$ is a geometric mean of J_i^0 and J_j^0.

EQeq+C

Bond-order-corrected Extended Charge Equilibration Method [18] follows exactly the same procedure as EQeq, however, after the computation is done, some corrections are added to the original charges, i.e.:

$$q_i = q_i^0 + \sum_{j \neq i} T_{i,j} B_{i,j} \tag{41}$$

where q_i^0 is original charge from EQeq, and

$$T_{i,j} = D_i - D_j \tag{42}$$

$$B_{i,j} = \exp\left[-\alpha \left(R_{i,j} - r_i - r_j\right)\right] \tag{43}$$

where D is an atom parameter, α is a common parameter and r stands for covalent radius.

Complexity of implemented approaches

The following table summarizes information about computational costs of the implemented methods, both in terms of time and memory complexity (expressed using $\mathcal{O}$ notation). The symbol N denotes the number of atoms, M is the number of bonds.

Method	Time	Memory
DelRe	N^3	N^2
PEOE	$N + M$	N
Charge2	$N + M$	1
EEM*	N^3	N^2
MPEOE	$N + M$	N
QEq*	N^3	N^2
ABEEM	$(N + M)^3$	$(N + M)^2$
GDAC	$N + M$	N
MGC	N^3	N^2
SFKEEM*	N^3	N^2
KCM	N^3	N^2
DENR	N^3	N^2
TSEF	N^3	N^2
SMP/QEq*	N^3	N^2
VEEM	N	1
EQeq*	N^3	N^2
EQeq+C*	N^3	N^2

Methods marked with * can utilize *cutoff* and *cover* complexity reductions described in the next section.

Assessment of charges quality

In order to compare two sets of charges, two descriptors are commonly used. The first one is the Pearson's correlation coefficient (R); the second one is the root mean square deviation (RMSD). In the following sections, we denote the charge sets as $X = (x_1, \ldots, x_n)$ and $Y = (y_1, \ldots, y_n)$, $\overline{x}$ and $\overline{y}$ represent the arithmetic means of x_i and y_i values, respectively.

Pearson's correlation coefficient (R)

Pearson's correlation coefficient describes the linear dependence between two sets of values. Its value ranges from -1 (negative correlation) through 0 (no correlation) to 1 (positive correlation). Sometimes, the value of squared Pearson's correlation coefficient (R^2) is used.

$$R(X,Y) = \frac{\sum_{i=1}^{n}(x_i - \overline{x}) \cdot (y_i - \overline{y})}{\sqrt{\sum_{i=1}^{n}(x_i - \overline{x})^2} \cdot \sqrt{\sum_{i=1}^{n}(y_i - \overline{y})^2}}$$

Root mean square deviation (RMSD)

Root mean square deviation is another measure which can be used to compare two sets of values. When RMSD is zero, the sets are identical. Otherwise, a smaller value indicates higher similarity.

$$\mathrm{RMSD}(X,Y) = \sqrt{\frac{1}{n}\sum_{i=1}^{n}(x_i - y_i)^2}$$

Cutoff and cover approaches

Solving the electronegativity equalization system of linear equations can be troublesome for very large structures as it requires, in general, $\mathcal{O}(N^3)$ steps and $\mathcal{O}(N^2)$ memory. The original AtomicChargeCalculator (ACC) introduced two *divide and conquer* complexity reduction algorithms to overcome this issue. Since ACC only supports EEM, these approaches were called *EEM Cutoff* and *EEM Cover*. ACC II extended these approaches to other applicable methods, referencing them simply as *cutoff* and *cover*.

Here, we provide the description of these approaches as stated in the ACC publication's [19] Additional file 1:

EEM Cutoff

For each atom in the molecule, ACC generates a fragment made up of all atoms within a cutoff radius R of the original atom. The values of the inter-atomic distances and EEM parameters are obtained in the same way as when solving the full EEM matrix. The total fragment charge Q_F is a quota of the total molecular charge Q, proportional to the number of atoms in the fragment (N_F), and irrespective of the nature of these atoms:

$$Q_F = \frac{Q \cdot N_F}{N}$$

Then ACC solves the EEM matrix equation for this fragment, and returns the charge for the atom used when generating the fragment. The same procedure is applied for all fragments, obtaining a set of charges for all the atoms in the molecule. Then, each atomic charge q_i is corrected by the addition of:

$$\frac{Q - \sum_{i=1}^{N} q_i}{N}$$

so that the sum of all atomic charges equals the total molecular charge Q.

EEM Cover

The EEM Cover approach builds on the principles of EEM Cutoff to split the EEM matrix into smaller matrices. However, EEM Cover generates fragments only for a subset of atoms in the molecule. The procedure selects fragment-generating atoms so that: (i) no two such atoms are connected to each other, and (ii) each atom in the molecule has at least one neighbor (within two bonds) which was selected. This procedure ensures that each atom in the molecule will eventually contribute to at least one fragment, and thus the entire volume of the molecule is covered. ACC solves the EEM matrix equation for each fragment, and returns a list of charge contributions for all atoms encountered in the calculations. The charge on each atom in the molecule is then computed as the sum of its charge contributions from all fragments where the atom is present. Further, each atomic charge q_i is corrected by the addition of:

$$\frac{Q - \sum_{i=1}^{N} q_i}{N}$$

so that the sum of all atomic charges equals the total molecular charge Q.

Time and space complexity

For a given sphere radius R, these approaches effectively reduce the time and space complexity to $\mathcal{O}(R^6 N + R^2 N \log N)$ and $\mathcal{O}(R^4 N + N \log N)$, respectively. [19]

Accuracy

Employing these approximative schemes may introduce some loss of accuracy when comparing with the original charges. Their assessment was made for original ACC for EEM [19] or in [20] where author states that "according to tests, the EEM Cover method produces results that are practically identical to solving the EEM matrix for the full original system". Here, we present a simplified[1] version of the table taken from [20]:

PDB ID	# of atoms	Total charge	R	RMSD
1lfg	5884	0	0.9998	0.0080
		-10	0.9999	0.0064
		8	0.9997	0.0094
35tu	13934	0	0.9999	0.0039
		-10	1.0000	0.0034
		8	0.9999	0.0045
1tye	21013	0	1.0000	0.0033
		-10	1.0000	0.0030
		8	0.9999	0.0037
1aoc	28708	0	1.0000	0.0021
		-10	1.0000	0.0019
		8	1.0000	0.0024

Table 1: Comparison of Full EEM vs. EEM Cover method for computing partial atomic charges. Parameters EX-NPA_6-31Gd_gas [21] were used in the computation.

[1]Only for $R = 12$ Å, which is used in ACC II.

Notes on the implementation

Following section presents some notes and discusses differences in implementation of the some methods compared to the description used in original publication.

GDAC

Only a subset of the parameters is used.

QEq

The scheme is not iterative, expression for $J_{i,j}$ is taken from [22] as in SMP/QEq:

$$J_{i,j} = \left(\frac{1}{(2\sqrt{B_i B_j})^3} + R_{i,j}^3 \right)^{-1/3} \tag{44}$$

EQeq and EQeq+C

Only the non-periodic case without non-zero charge centers is supported.

Applications of charges

Partial atomic charges, obtained by empirical charge calculation methods, can be applied for example in the following fields:

- descriptors for QSAR and QSPR modelling [23, 24, 25, 26, 27, 28, 29, 30, 31]
- pharmacophore design [32, 33, 34]
- virtual screening [35, 36, 37]
- molecular docking [38, 39, 40]
- similarity searches [41, 42, 43]
- conformers generation [44]
- molecular dynamics [7, 45, 46, 47]
- study of mechanisms of chemical actions [48, 40, 49]

Bibliography

[1] Giuseppe Del Re. "812. A simple MO–LCAO method for the calculation of charge distributions in saturated organic molecules". In: *Journal of the Chemical Society (Resumed)* (1958), pp. 4031–4040.

[2] Johann Gasteiger and Mario Marsili. "A new model for calculating atomic charges in molecules". In: *Tetrahedron Letters* 19.34 (1978), pp. 3181–3184.

[3] Johann Gasteiger and Mario Marsili. "Iterative partial equalization of orbital electronegativity—a rapid access to atomic charges". In: *Tetrahedron* 36.22 (1980), pp. 3219–3228.

[4] Raymond J Abraham, Lee Griffiths, and Philip Loftus. "Approaches to charge calculations in molecular mechanics". In: *Journal of Computational Chemistry* 3.3 (1982), pp. 407–416.

[5] Wilfried J Mortier, Swapan K Ghosh, and S Shankar. "Electronegativity-equalization method for the calculation of atomic charges in molecules". In: *Journal of the American Chemical Society* 108.15 (1986), pp. 4315–4320.

[6] Kyoung Tai No, J Andrew Grant, and Harold A Scheraga. "Determination of net atomic charges using a modified partial equalization of orbital electronegativity method. 1. Application to neutral molecules as models for polypeptides". In: *Journal of Physical Chemistry* 94.11 (1990), pp. 4732–4739.

[7] Anthony K Rappe and William A Goddard III. "Charge equilibration for molecular dynamics simulations". In: *The Journal of Physical Chemistry* 95.8 (1991), pp. 3358–3363.

[8] Zhong-Zhi Yang and Chang-Sheng Wang. "Atom- bond electronegativity equalization method. 1. Calculation of the charge distribution in large molecules". In: *The Journal of Physical Chemistry A* 101.35 (1997), pp. 6315–6321.

[9] Kwang-Hwi Cho et al. "A fast method for calculating geometry-dependent net atomic charges for polypeptides". In: *The Journal of Physical Chemistry B* 105.17 (2001), pp. 3624–3634.

[10] AA Oliferenko et al. "A new topological model for the calculation of partial atomic charges". In: *Doklady Chemistry*. Vol. 375. 4-6. Springer. 2000, pp. 281–284.

[11] Alexander A Oliferenko et al. "Novel point charge models: reliable instruments for molecular electrostatics". In: *Journal of Physical Organic Chemistry* 14.6 (2001), pp. 355–369.

[12] J Chaves et al. "Toward an alternative hardness kernel matrix structure in the Electronegativity Equalization Method (EEM)". In: *Journal of chemical information and modeling* 46.4 (2006), pp. 1657–1665.

[13] Olexander Yakovenko et al. "Kirchhoff atomic charges fitted to multipole moments: implementation for a virtual screening system". In: *Journal of computational chemistry* 29.8 (2008), pp. 1332–1343.

[14] DA Shulga et al. "Fast tools for calculation of atomic charges well suited for drug design". In: *SAR and QSAR in Environmental Research* 19.1-2 (2008), pp. 153–165.

[15] Min Zhang and René Fournier. "Self-Consistent Charge Equilibration Method and Its Application to Au13Na n (n= 1, 10) Clusters". In: *The Journal of Physical Chemistry A* 113.13 (2009), pp. 3162–3170.

[16] Ya-xin Wu, Chen-zhong Cao, and Hua Yuan. "Equalized electronegativity based on the valence electrons and its application". In: *Chinese Journal of Chemical Physics* 24.1 (2011), p. 31.

[17] Christopher E Wilmer, Ki Chul Kim, and Randall Q Snurr. "An extended charge equilibration method". In: *The journal of physical chemistry letters* 3.17 (2012), pp. 2506–2511.

[18] Geoffrey C Martin-Noble et al. "EQeq+ C: an empirical bond-order-corrected extended charge equilibration method". In: *Journal of chemical theory and computation* 11.7 (2015), pp. 3364–3374.

[19] Crina-Maria Ionescu et al. "AtomicChargeCalculator: interactive web-based calculation of atomic charges in large biomolecular complexes and drug-like molecules". In: *Journal of cheminformatics* 7.1 (2015), p. 50.

[20] David Sehnal. "Methods for the Analysis of 3D Structural Fragments in Biomolecules [online]". Doctoral theses, Dissertations. Masaryk University, Faculty of Informatics, Brno, 2015 [cit. 2020-04-23]. URL: https://is.muni.cz/th/ri9hp/.

[21] Crina-Maria Ionescu et al. "Rapid calculation of accurate atomic charges for proteins via the electronegativity equalization method". In: *Journal of chemical information and modeling* 53.10 (2013), pp. 2548–2558.

[22] Jaap N Louwen and Eelco TC Vogt. "Semi-empirical atomic charges for use in computational chemistry of molecular sieves". In: *Journal of Molecular Catalysis A: Chemical* 134.1-3 (1998), pp. 63–77.

[23] Radka Svobodová Vařeková et al. "Predicting pKa values of substituted phenols from atomic charges: comparison of different quantum mechanical methods and charge distribution schemes". In: *Journal of chemical information and modeling* 51.8 (2011), pp. 1795–1806.

[24] Radka Svobodová Vařeková et al. "Predicting pK a values from EEM atomic charges". In: *Journal of cheminformatics* 5.1 (2013), p. 18.

[25] Stanislav Geidl et al. "How does the methodology of 3D structure preparation influence the quality of p K a prediction?" In: *Journal of chemical information and modeling* 55.6 (2015), pp. 1088–1097.

[26] Steven L Dixon and Peter C Jurs. "Estimation of pKa for organic oxyacids using calculated atomic charges". In: *Journal of computational chemistry* 14.12 (1993), pp. 1460–1467.

[27] Jinhua Zhang, Thomas Kleinöder, and Johann Gasteiger. "Prediction of pKa values for aliphatic carboxylic acids and alcohols with empirical atomic charge descriptors". In: *Journal of chemical information and modeling* 46.6 (2006), pp. 2256–2266.

[28] Kevin C Gross, Paul G Seybold, and Christopher M Hadad. "Comparison of different atomic charge schemes for predicting pKa variations in substituted anilines and phenols". In: *International journal of quantum chemistry* 90.1 (2002), pp. 445–458.

[29] TARAVAT GHAFOURIAN and JOHN C DEARDEN. "The Use of Atomic Charges and Orbital Energies as Hydrogen-bonding-donor Parameters for QSAR Studies: Comparison of MNDO, AM1 and PM3 Methods". In: *Journal of pharmacy and pharmacology* 52.6 (2000), pp. 603–610.

[30] Arkadiusz Z Dudek, Tomasz Arodz, and Jorge Gálvez. "Computational methods in developing quantitative structure-activity relationships (QSAR): a review". In: *Combinatorial chemistry & high throughput screening* 9.3 (2006), pp. 213–228.

[31] Mati Karelson, Victor S Lobanov, and Alan R Katritzky. "Quantum-chemical descriptors in QSAR/QSPR studies". In: *Chemical reviews* 96.3 (1996), pp. 1027–1044.

[32] Roberto Todeschini and Viviana Consonni. *Handbook of molecular descriptors*. Vol. 11. John Wiley & Sons, 2008.

[33] Jorge Galvez et al. "Charge indexes. New topological descriptors". In: *Journal of Chemical Information and Computer Sciences* 34.3 (1994), pp. 520–525.

[34] Dietmar Stalke. "Meaningful structural descriptors from charge density". In: *Chemistry–A European Journal* 17.34 (2011), pp. 9264–9278.

[35] Raimund Mannhold, Hugo Kubinyi, and Gerd Folkers. *Pharmacophores and pharmacophore searches*. Vol. 32. John Wiley & Sons, 2006.

[36] Preston J MacDougall and Christopher E Henze. "Fleshing-out Pharmacophores with Volume Rendering of the Laplacian of the Charge Density and Hyperwall Visualization Technology". In: *The quantum theory of atoms in molecules: from solid state to DNA and drug design* (2007), pp. 499–514.

[37] OO Clement and AT Mehl. “HipHop: pharmacophores based on multiple common-feature alignments”. In: *Pharmacophore perception, development, and use in drug design* (2000), pp. 69–84.

[38] Hwangseo Park, Jinuk Lee, and Sangyoub Lee. “Critical assessment of the automated AutoDock as a new docking tool for virtual screening”. In: *Proteins: Structure, Function, and Bioinformatics* 65.3 (2006), pp. 549–554.

[39] Benjamin Nebgen et al. “Transferable dynamic molecular charge assignment using deep neural networks”. In: *Journal of chemical theory and computation* 14.9 (2018), pp. 4687–4698.

[40] Hrvoje Rimac et al. “Warfarin and flavonoids do not share the same binding region in binding to the IIA subdomain of human serum albumin”. In: *Molecules* 22.7 (2017), p. 1153.

[41] Simon K Kearsley et al. “Chemical similarity using physiochemical property descriptors”. In: *Journal of Chemical Information and Computer Sciences* 36.1 (1996), pp. 118–127.

[42] Nina Nikolova and Joanna Jaworska. “Approaches to measure chemical similarity–a review”. In: *QSAR & Combinatorial Science* 22.9-10 (2003), pp. 1006–1026.

[43] John D Holliday et al. “Calculation of intersubstituent similarity using R-group descriptors”. In: *Journal of chemical information and computer sciences* 43.2 (2003), pp. 406–411.

[44] Mikko J Vainio and Mark S Johnson. “Generating conformer ensembles using a multiobjective genetic algorithm”. In: *Journal of chemical information and modeling* 47.6 (2007), pp. 2462–2474.

[45] Kimberly Chenoweth, Adri CT Van Duin, and William A Goddard. “ReaxFF reactive force field for molecular dynamics simulations of hydrocarbon oxidation”. In: *The Journal of Physical Chemistry A* 112.5 (2008), pp. 1040–1053.

[46] Marjan A Nejad and Herbert M Urbassek. “Insulin adsorption on functionalized silica surfaces: an accelerated molecular dynamics study”. In: *Journal of molecular modeling* 24.4 (2018), p. 89.

[47] Jong Wha Lee et al. “Structural characterization of small molecular ions by ion mobility mass spectrometry in nitrogen drift gas: improving the accuracy of trajectory method calculations”. In: *Analyst* 143.8 (2018), pp. 1786–1796.

[48] Crina-Maria Ionescu et al. “Charge profile analysis reveals that activation of pro-apoptotic regulators Bax and Bak relies on charge transfer mediated allosteric regulation”. In: *PLoS computational biology* 8.6 (2012).

[49] Simon Wheeler, Ralf Schmid, and Dan J Sillence. “Lipid–protein interactions in Niemann–pick type C disease: insights from molecular modeling”. In: *International journal of molecular sciences* 20.3 (2019), p. 717.
