# Supplementary material for: Atomic Charge Calculator II: web-based tool for the calculation of partial atomic charges
Source: Nucleic Acids Res. 2020 May 13;48(W1):W591–6. doi: 10.1093/nar/gkaa367 (PMC7319571; doi:10.1093/nar/gkaa367)
Supplement: gkaa367_Supplemental_Files [file gkaa367_supplemental_files.zip › Supplementary table 1.pdf]

Supplementary table 1: Citation data of partial atomic charge calculation methods that have been mentioned in the article. Data were obtained on 31st March 2020 from the Web of Science citation database ("All Databases" dataset) that can be accessed at https://www.webofknowledge.com/.

**Method**	**Publication**					**Total method cit. count**
	**Year**	**Author(s)**	**Journal**	**Cit. count**	**Ref.**	
DelRe	1958	Del Re, G.	J. Chem. Soc.	531	[1]	531
PEOE	1978	Gasteiger, J. and Marsili, M.	Tetrahedron Lett.	254	[2]	
	1980	Gasteiger, J. and Marsili, M.	Tetrahedron	3,104	[3]	3,358
Charge2	1982	Abraham, R.J. and Hudson, B.	J. Comput. Chem.	68	[4]	68
EEM	1986	Mortier, W.J., et. al.	J. Am. Chem. Soc.	722	[5]	722
MPEOE	1990	No, K.T., et. al.	J. Phys. Chem.	118	[6]	118
QEq	1991	Rappé, A.K. and Goddard, W.A.	J. Phys. Chem.	2,301	[7]	2,301
ABEEM	1997	Yang, Z.-Z. and Wang, C.-S.	J. Phys. Chem. A	154	[8]	154
GDAC	2001	Cho, K.H., et. al.	J. Phys. Chem. B	24	[9]	24
MGC	2000	Oliferenko, A.A., et. al.	Dokl. Chem.	6	[10]	
	2001	Oliferenko, A.A., et. al.	J. Phys. Org. Chem.	17	[11]	23
SFKEEM	2006	Chaves, J., et. al.	J. Chem. Inf. Model.	12	[12]	12
KCM	2008	Yakovenko, O., et. al.	J. Comput. Chem.	19	[13]	19
DENR	2008	Shulga, D.A., et. al.	SAR QSAR Environ. Res.	10	[14]	10
TSEF	2008	Shulga, D.A., et. al.	SAR QSAR Environ. Res.	10	[14]	10
SMP/QEq	2009	Zhang, M. and Fournier, R.	J. Phys. Chem. A	12	[15]	12
VEEM	2011	Wu, Y.-X., et. al.	Chinese J. Chem. Phys.	4	[16]	4
EQeq	2012	Wilmer, C.E., et. al.	J. Phys. Chem. Lett.	117	[17]	117
EQeq+C	2015	Martin-Noble, G.C., et. al.	J. Chem. Theory Comput.	8	[18]	8

References

[1] Giuseppe Del Re. "812. A simple MO–LCAO method for the calculation of charge distributions in saturated organic molecules". In: *Journal of the Chemical Society (Resumed)* (1958), pp. 4031–4040.

[2] Johann Gasteiger and Mario Marsili. "A new model for calculating atomic charges in molecules". In: *Tetrahedron Letters* 19.34 (1978), pp. 3181–3184.

[3] Johann Gasteiger and Mario Marsili. "Iterative partial equalization of orbital electronegativity—a rapid access to atomic charges". In: *Tetrahedron* 36.22 (1980), pp. 3219–3228.

[4] Raymond J Abraham, Lee Griffiths, and Philip Loftus. "Approaches to charge calculations in molecular mechanics". In: *Journal of Computational Chemistry* 3.3 (1982), pp. 407–416.

[5] Wilfried J Mortier, Swapan K Ghosh, and S Shankar. "Electronegativity-equalization method for the calculation of atomic charges in molecules". In: *Journal of the American Chemical Society* 108.15 (1986), pp. 4315–4320.

[6] Kyoung Tai No, J Andrew Grant, and Harold A Scheraga. "Determination of net atomic charges using a modified partial equalization of orbital electronegativity method. 1. Application to neutral molecules as models for polypeptides". In: *Journal of Physical Chemistry* 94.11 (1990), pp. 4732–4739.

[7] Anthony K Rappe and William A Goddard III. "Charge equilibration for molecular dynamics simulations". In: *The Journal of Physical Chemistry* 95.8 (1991), pp. 3358–3363.

[8] Zhong-Zhi Yang and Chang-Sheng Wang. "Atom- bond electronegativity equalization method. 1. Calculation of the charge distribution in large molecules". In: *The Journal of Physical Chemistry A* 101.35 (1997), pp. 6315–6321.

[9] Kwang-Hwi Cho et al. "A fast method for calculating geometry-dependent net atomic charges for polypeptides". In: *The Journal of Physical Chemistry B* 105.17 (2001), pp. 3624–3634.

[10] AA Oliferenko et al. "A new topological model for the calculation of partial atomic charges". In: *Doklady Chemistry*. Vol. 375. 4-6. Springer. 2000, pp. 281–284.

[11] Alexander A Oliferenko et al. "Novel point charge models: reliable instruments for molecular electrostatics". In: *Journal of Physical Organic Chemistry* 14.6 (2001), pp. 355–369.

[12] J Chaves et al. "Toward an alternative hardness kernel matrix structure in the Electronegativity Equalization Method (EEM)". In: *Journal of chemical information and modeling* 46.4 (2006), pp. 1657–1665.

[13] Olexander Yakovenko et al. "Kirchhoff atomic charges fitted to multipole moments: implementation for a virtual screening system". In: *Journal of computational chemistry* 29.8 (2008), pp. 1332–1343.

[14] DA Shulga et al. "Fast tools for calculation of atomic charges well suited for drug design". In: *SAR and QSAR in Environmental Research* 19.1-2 (2008), pp. 153–165.

[15] Min Zhang and René Fournier. "Self-Consistent Charge Equilibration Method and Its Application to Au13Na n (n= 1, 10) Clusters". In: *The Journal of Physical Chemistry A* 113.13 (2009), pp. 3162–3170.

[16] Ya-xin Wu, Chen-zhong Cao, and Hua Yuan. "Equalized electronegativity based on the valence electrons and its application". In: *Chinese Journal of Chemical Physics* 24.1 (2011), p. 31.

[17] Christopher E Wilmer, Ki Chul Kim, and Randall Q Snurr. "An extended charge equilibration method". In: *The journal of physical chemistry letters* 3.17 (2012), pp. 2506–2511.

[18] Geoffrey C Martin-Noble et al. "EQeq+ C: an empirical bond-order-corrected extended charge equilibration method". In: *Journal of chemical theory and computation* 11.7 (2015), pp. 3364–3374.
