# Supplementary material for: Atomic Charge Calculator II: web-based tool for the calculation of partial atomic charges
Source: Nucleic Acids Res. 2020 May 13;48(W1):W591–6. doi: 10.1093/nar/gkaa367 (PMC7319571; doi:10.1093/nar/gkaa367)
Supplement: gkaa367_Supplemental_Files [file gkaa367_supplemental_files.zip › Supplementary table 2.pdf]

Supplementary table 2: List of all parameters included in ACC II.

Method	Parameters name	Publication DOI
ABEEM	Yang 1997	10.1021/jp9711048
Charge2	Abraham 1982	10.1002/jcc.540030316
DelRe	Del Re 1958	10.1039/JR9580004031
DENR	Rappe 1991	10.1021/j100161a070
EEM	Baekelandt 1991	10.1021/ja00018a003
EEM	Bultinck 2002 (CHELPG)	10.1021/jp020547v
EEM	Bultinck 2002 (Hirshfeld)	10.1021/jp020547v
EEM	Bultinck 2002 (MK)	10.1021/jp020547v
EEM	Bultinck 2002 (Mulliken)	10.1021/jp020547v
EEM	Bultinck 2002 (NPA)	10.1021/jp020547v
EEM	Bultinck 2004 (AIM)	10.1021/jp046928l
EEM	Geidl 2015 (Cheminf_b3lyp_aim)	10.1186/s13321-015-0107-1
EEM	Geidl 2015 (Cheminf_b3lyp_mpa)	10.1186/s13321-015-0107-1
EEM	Geidl 2015 (Cheminf_b3lyp_npa)	10.1186/s13321-015-0107-1
EEM	Geidl 2015 (Cheminf_hf_aim)	10.1186/s13321-015-0107-1
EEM	Geidl 2015 (Cheminf_hf_mpa)	10.1186/s13321-015-0107-1
EEM	Geidl 2015 (Cheminf_hf_npa)	10.1186/s13321-015-0107-1
EEM	Ionescu 2013 (EX-MPA_6-31Gd_PCM)	10.1021/ci400448n
EEM	Ionescu 2013 (EX-MPA_6-31Gd_gas)	10.1021/ci400448n
EEM	Ionescu 2013 (EX-NPA_6-31Gd_PCM)	10.1021/ci400448n
EEM	Ionescu 2013 (EX-NPA_6-31Gd_gas)	10.1021/ci400448n
EEM	Ouyang 2009 (124 calibrated set)	10.1039/b821696g
EEM	Ouyang 2009 (131 calibrated set)	10.1039/b821696g
EEM	Ouyang 2009 (hybridization-dependent)	10.1039/b821696g
EEM	Racek 2016 (ccd2016_mpa)	10.1186/s13321-016-0171-1
EEM	Racek 2016 (ccd2016_mpa2)	10.1186/s13321-016-0171-1
EEM	Racek 2016 (ccd2016_npa)	10.1186/s13321-016-0171-1
EEM	Racek 2016 (ccd2016_npa2)	10.1186/s13321-016-0171-1
EEM	Svobodova 2007 (cbeg2)	10.3390/i8070572
EEM	Svobodova 2007 (chal2)	10.3390/i8070572
EEM	Svobodova 2007 (chm2)	10.3390/i8070572
EEM	Svobodova 2007 (cmet2)	10.3390/i8070572
EQeq+C	Martin-Noble 2015 (ATMO/H-I)	10.1021/acs.jctc.5b00037
EQeq+C	Martin-Noble 2015 (MOF/REPEAT)	10.1021/acs.jctc.5b00037
GDAC	Cho 2001	10.1021/jp0023213
KCM	Yakovenko 2008 (initial)	10.1007/BFb0029840
MPEOE	No 1990 (DP1)	10.1021/j100374a066
PEOE	Gasteiger 1980	10.1016/0040-4020(80)80168-2
QEq	Rappe 1991	10.1021/j100161a070
SFKEEM	Chaves 2006	10.1021/ci050505e
SMP/QEq	Zhang 2009	10.1021/jp8063273
TSEF	Rappe 1991	10.1021/j100161a070
